# Probiotic Activity of *Enterococcus faecium* and *Lactococcus lactis* Isolated from Thai Fermented Sausages and Their Protective Effect Against *Clostridium difficile*

**DOI:** 10.1007/s12602-019-09536-7

**Published:** 2019-03-19

**Authors:** Panya Dowdell, Surang Chankhamhaengdecha, Watanalai Panbangred, Tavan Janvilisri, Amornrat Aroonnual

**Affiliations:** 1 Department of Biochemistry, Faculty of Science, Mahidol University, Bangkok, Thailand; 2 Department of Biology, Faculty of Science, Mahidol University, Bangkok, Thailand; 3 Department of Biotechnology, Faculty of Science, Mahidol University, Bangkok, Thailand; 4 Department of Tropical Nutrition and Food Science, Faculty of Tropical Medicine, Mahidol University, Bangkok, 10400 Thailand

**Keywords:** Probiotics, *Lactococcus lactis*, *Enterococcus faecium*, LAB, *Clostridium difficile*

## Abstract

Lactic acid bacteria, *Enterococcus faecium* and *Lactococcus lactis*, previously isolated from Thai fermented sausages were elucidated their probiotic properties especially in the control of *Clostridium difficile* 630. Both isolates survived in simulated gastric solution at pH 3 followed in simulated intestinal solution at pH 8. The presence of skimmed milk also helped the bacteria to survive through acidic and alkaline in gastrointestinal conditions. The adhesion properties of both isolates were tested using a human colon adenocarcinoma cell line. The result showed that both isolates exhibited desirable probiotic properties which adhered to Caco-2 cells. The neutralized cell-free supernatant of both isolates demonstrated that no cytotoxicity toward Caco-2 cells vice versa cell-free supernatant of *C. difficile* 630 toward Caco-2 cell demonstrated high toxicity. The immunomodulation effect in response to bacterial neutralized cell-free supernatant and cell-free supernatant was also studied. The expression level of pro-inflammatory cytokine of Caco-2 cell which are tumor necrosis factor-α and interleukin-8 was evaluated using quantitative reverse transcriptase PCR. Both isolates were able to diminish the expression level of TNF-α and IL-8 induced by the cell-free supernatant of *C. difficile* 630. Hence, these isolates would be able to improve the gut health through counteracting the *C. difficile*-associated intestinal inflammation in human cell lines. These results may contribute to the development of the isolates using as probiotics.

## Introduction

Lactic acid bacteria (LAB) have been widely used in the food manufacturing industry, primarily for fermentation processes. They are now frequently used as probiotics, bacteria that promote better health and well-being. Their proposed mode of action is referred to as colonization resistance, the creation of a physical barrier which enhances the integrity of tight junctions, and preventing the entry of pathogens. They possess antimicrobial activity via the production of antimicrobial peptides known as bacteriocins. They can also stimulate the production of anti-inflammatory cytokines, and inhibit or trigger an innate immune response by initiating TNF-α production to recruit neutrophils to the site of infection [1, 2].

*Clostridium difficile* is a Gram-positive, spore-forming anaerobic bacterium. It is one of the major causes of nosocomial diarrhea, ranging from mild diarrhea to life-threatening pseudomembranous colitis. The organism produces two major virulence toxins known as *C. difficile* toxin A (TcdA) and *C. difficile* toxin B (TcdB) that play crucial roles in the disruption of the actin cytoskeleton and the impairment of tight junctions in the intestinal epithelial cells, resulting in extensive damage to the large intestine [3–5]. *C. difficile* is thought to be a multidrug-resistant pathogen; due to its broad-spectrum antibiotic resistance, different alternative methodologies—including the use of probiotic bacteria such as LAB—have been investigated for the prevention and treatment of *C. difficile* infection (CDI).

Previous studies have reported the effects of LAB against *C. difficile*. Lactic acid produced by *Streptococcus thermophilus* inhibited the growth of *C. difficile* in a dose-dependent manner and decreased gene expression of TdcA [6]. Another study demonstrated that *Lactobacillus rhamnosus* GG displayed inhibitory effects on *C. difficile* growth and protected kidney epithelial cells from African green monkey (Vero cells) from toxigenic *C. difficile* [7]. Various strains of LAB such as *L. rhamnosus* LR5, *Lactococcus lactis* SL3, *Bifidobacterium breve* BR3, and *Bifidobacterium lactis* BL3 have demonstrated antimicrobial activity by inhibiting the growth of *C. difficile* [8].

In the present study, we aim to elucidate the probiotic properties of *E. faecium* and *L. lactis* isolated from Thai fermented sausages that demonstrated antimicrobial activity via the production of bacteriocins [9]. The properties include the ability to survive in simulated gastrointestinal solutions, adhesive properties to epithelium cell, cytotoxicity, and immunomodulation against multi-drug resistant *C. difficile* strain 630 (CD630) which is PCR-ribotype 012, PCR ribotyping of clinically important hypervirulent strain of *C. difficile*. The results will provide essential information regarding the use of these bacteria as probiotics.

## Materials and Methods

### Strains and Bacterial Culture Conditions

*E. faecium* and *L. lactis* producing bacteriocins, such as enterocin and nisin, respectively, were isolated from Thai fermented sausages [9]. Briefly, Thai fermented sausage samples were bought from local market, and samples were mixed with Criterion™ Lactobacilli de Man, Rogosa, and Sharpe (MRS) broth (Hardy Diagnostics, Santa Maria, CA, USA). After incubation at 37 °C for 1 h with continuous shaking, the samples were tenfold serially diluted and the appropriated dilutions were then spread onto MRS agar. The plate was then incubated at 37 °C for 24 h. The strains were identified by determination of 16S ribosomal RNA gene using universal primers for 16S rDNA of bacteria [10].

The bacteria were maintained on MRS agar at 37 °C for 24 h. The single colony of LAB was inoculated into MRS broth and further incubated at 37 °C for 24 h under aerobic condition. CD630, multi-drug resistant strain, was kindly provided by Prof. Nigel Minton, University of Nottingham, UK, and was streaked on Brain Heart Infusion (BHI) agar (Himedia, Mumbai, India) and incubated anaerobically at 37 °C for 24 h to obtain single colonies. BHI broth supplemented with 0.1% sodium taurocholate (TCI, Chuo-ku, Tokyo, Japan) and 0.5% yeast extract (BD, Franklin Lakes, NJ, USA) was inoculated with single colonies of CD630 and incubated under anaerobic condition for use in subsequent tests. For long-term storage, overnight cultures of each strain were transferred into sterilized glycerol to obtain a final concentration of 20% and kept at − 80 °C.

### Preparation of the NCFS of LAB and CFS of CD630

Fifty milliliters of MRS broth was inoculated with 1% (*v*/*v*) of *E. faecium* and *L. lactis* culture as described above. The cultures were incubated at 37 °C for 16–18 h under aerobic condition. Bacterial supernatant was collected after centrifugation at 8000×*g* at 4 °C for 10 min and were neutralized using 1 M NaOH following by filtration through a sterilized 0.22-μm filter (Merck, Kenilworth, NJ, USA) to obtain neutralized cell-free supernatant (NCFS). CD630 was cultivated in BHI broth as mentioned earlier. Fifty milliliters of BHI broth was inoculated with 1% (*v*/*v*) of CD630 culture and further incubated at 37 °C for 24 h anaerobically. CD630 supernatant was also collected and filtered as described previously to obtain cell-free supernatant (CFS).

Total protein of the NCFS of *E. faecium* and *L. lactis* isolates and the CFS of CD630 were measured using Bradford solution (Bio-Rad, Hercules, CA, USA) following the manufacturer’s protocol. Bovine serum albumin (Takara, Kusatsu-ku, Shiga, Japan) was used as a standard protein. The optical density (OD) was measured at *λ* = 595 nm using UV1800 Spectrophotometer (Shimadzu, Nakagyo-ku, Kyoto, Japan).

## Assay of Probiotics Properties

### Survival of LAB in Simulated Gastrointestinal Solution

The growth of *L. lactis* and *E. faecium* isolates in the presence of simulated gastrointestinal fluid is elucidated. Ten milliliters of each isolate culture was collected by centrifugation at 8000×*g* at 4 °C for 10 min. Cell pellets were then washed twice with phosphate-buffered saline (PBS) at pH 7.4, followed by re-suspension in 50 ml of simulated gastric solutions (125 mM NaCl, 7 mM KCl, 45 mM NaHCO_3_, and 3 g/l pepsin; pH 2 and pH 3). To elucidate the effect of a food matrix on the isolates during gastric transit, the survival of both isolates in simulated gastric solution at pH 2 in the presence of skimmed milk (11% *w*/*v*) (Himedia, Mumbai, India) was also observed. All bacterial suspensions were then incubated at 37 °C with agitation. Samples were taken after 0, 90, and 180 min of incubation. After 180 min of incubation with simulated gastric solution, bacterial cells were harvested by centrifugation. Cell pellets were washed twice with PBS and re-suspended in simulated intestinal solution (0.1% *w*/*v* pancreatin (TCI, Chuo-ku, Tokyo, Japan) and 0.15% *w*/*v* oxgall bile salts (Neogen, Lansing, MI, USA); pH 8.0). The bacterial suspensions were continually incubated as described above. Samples of the bacterial suspensions were taken at 0, 90, and 180 min after incubation with simulated intestinal solution. Tenfold serial dilutions in PBS of each bacterial suspension were applied, and appropriated dilutions were spread on MRS agar plates. Cell viability was enumerated by counting the number of colony-forming units (CFU/ml) following overnight incubation at 37 °C [11].

### Adhesion Assay

Human colon adenocarcinoma cell line Caco-2 cells (ATCC®HTB-37™) were cultured in Hyclone™ Dulbecco’s modified Eagle’s medium (DMEM) (GE Healthcare-Bioscience, Pittsburgh, PA, USA) supplemented with 10% Gibco™ Fetal Bovine Serum (FBS) (Thermo Fisher Scientific, Waltham, MA, USA) and 1% of Hyclone™ Penicillin-Streptomycin 100× solution (GE Healthcare-Bioscience, Pittsburgh, PA, USA) at 37 °C with 5% CO_2_ and humidity.

Caco-2 cells were seeded in a 24-well plate and cultured for 14 days until cells differentiated. Five hundred microliters of appropriate bacterial dilution (10^5^ CFU/ml) were added to each well with a ratio of 10:1 (bacterial cells/Caco-2 cells) and incubated at 37 °C with 5% CO_2_ for 2 h. Cells were then washed with PBS thrice. Following the final wash, 500 μl of 0.1% Triton x-100 was added and pipetted vigorously to re-suspended Caco-2 cells with adhered bacterial cells. Tenfold serial dilutions of each mixture were done, and the appropriated dilutions were spread on MRS agar. Adhering bacteria were enumerated by counting CFU per milliliter following incubation at 37 °C for 24 h [12].

### Cytotoxicity Assay

Caco-2 cells were seeded in a 96-well plate (3 × 10^4^ cells/well) and incubated at 37 °C with 5% CO_2_ for 24 h. Medium was removed and 50 μl of DMEM was added to each well prior to the experiments. Fifty microliters of NCFS of *E. faecium* and *L. lactis* isolates and the CFS of CD630 with various total protein concentrations (100, 80, 40, 20, 10 μg/ml) were added into each well. The mixtures were incubated at 37 °C for 24 h. The mixtures were then removed, and 100 μl of 0.5 mg/ml of 3-(4,5-dimethylthiasol-2-yl)-2,5-diphenyltetrazolium bromide (MTT) solution (Merck, Kenilworth, NJ, USA) was added to each well, and the plate was further incubated for 2 h in darkness. The MTT solution was gently removed, 100 μl dimethyl sulfoxide (DMSO) (Merck, Kenilworth, NJ, USA) was added to solubilize formazan crystals, and the absorbance was measured at *λ* = 570 nm. DMEMs mixed with MRS or BHI were used as controls. Effects of LAB isolates against CD630 were also investigated under three different conditions: (1) coincubation: Caco-2 cells were incubated with 50 μl of 100 μg/ml NCFS of either *E. faecium* or *L. lactis* and 50 μl of 100 μg/ml CFS of CD630 following by incubation for 24 h; (2) protection: Caco-2 cells were pre-incubated with 50 μl of 100 μg/ml NCFS of either *E. faecium* or *L. lactis* for 2 h, followed by the addition of 50 μl of 100 μg/ml CFS of CD630, and the culture was further incubated for 24 h; and (3) prevention: Caco-2 cells were pre-incubated with 50 μl of 100 μg/ml CFS of CD630 for 2 h, followed by the addition of 50 μl of 100 μg/ml *E. faecium* or *L. lactis*, and further incubated for 24 h. DMEMs mixed with BHI and MRS were used as controls [13]. Experiments were conducted in triplicate. The Caco-2 cell viability was calculated using the following equation: % Cell viability = [(OD_sample_ × 100)/OD_control_].

### Immunofluorescent Imaging of Tight Junctions

Caco-2 cells were cultured in a 24-well plate (1 × 10^5^ cells/well) and incubated for 24 h. Cells were washed twice with PBS, and 500 μl of DMEM was added to each well followed by 500 μl of the NCFS of *E. faecium*, *L. lactis*, and the CFS of CD630 to a final concentration of 50 μg/ml total protein. Cells were incubated at 37 °C for 16 h, fixed with 4% *v*/*v* formaldehyde, permeabilized with 0.1% *v*/*v* Triton x-100, and washed thrice with PBS. Normal goat serum (5% *v*/*v*) (Merck, Kenilworth, NJ, USA) was used as a blocking solution. Primary antibodies against occludin (Merck, Kenilworth, NJ, USA) with the ratio of 1:100 were added, and cells were further incubated at 4 °C overnight. Cells were washed thrice with PBS, and FIT-C-conjugated secondary antibodies (1:100) (Merck, Kenilworth, NJ, USA) were added; cells were incubated at room temperature in darkness for 1 h. Cells were washed with PBS, followed by observation under a fluorescence microscope [14].

### RT-qPCR Analysis

Caco-2 cells were seeded in a 6-well plate (10^6^ cells/well) and cultured for 48 h. Five hundred microliters of DMEM was added to cells, followed by the addition of 500 μl of 100 μg/ml NCFS of *E. faecium*, *L. lactis*, or the CFS of CD630 into each well, and the plate was incubated for 16 h. The coincubation conditions, in which 500 μl of DMEM, 500 μl of 100 μg/ml NCFS of *E. faecium* or *L. lactis*, and 500 μl of 100 μg/ml CFS of CD630, were added to the same well, and were also prepared and incubated for 16 h. RNA extraction was performed using the illustra™ RNAspin Mini (GE Healthcare UK, Little Chalfont, Buckinghamshire, UK). Extracted RNA from all tested samples was quantified using NanoDrop Spectrophotometer at *λ* = 260 nm. To determine the purity of extracted RNA, the ratio of absorbance at *λ* = 260 and at *λ* = 280 nm (A260/A280) and the ratio of A260/A230 were also calculated. One microgram (1 μg) of each RNA sample was converted to cDNA using iScript™ Reverse Transcription Supermix for RT-qPCR (Bio-Rad, Hercules, CA, USA) following the manufacturer’s protocol and stored at − 20 °C until use. PCR was performed using 2× *Taq* Master Mix (Vivantis, Subang Jaya, Salangor Darul Ehsan, Malaysia) following the manufacturer’s protocol to obtain optimum conditions (Table 1). The expression level of IL-8 and TNF-α was investigated using Luna® Universal One-Step RT-qPCR Kit (NEB, Ipswich, MA, USA), while the expressions of β-actin were used as a control. The ΔΔCt method was used for determination of relative gene expression quantitation.

**Table 1:** Primer sequences and conditions used for PCR and RT-qPCR

Gene	Primer sequences	Expected product (bp)	PCR condition
β-Actin	F: 5′-GCACAGAGCCTCGCCTT-3′	112	94 °C for 2 min; 30 cycles of 94 °C 20s, 60 °C 2 min, 72 °C 30s: following by 72 °C for 7 min
	R: 5′-CCTTGCACATGCCGGAG-3′		
IL-8	F: 5′-AAGGTGCAGTTTTGCCAAGG-3′	204	94 °C for 2 min; 30 cycles of 94 °C 20s, 60 °C 2 min, 72 °C 30s: following by 72 °C for 7 min
	R: 5′-CAACCCTCTGCACCCAGTTT-3′		
TNF-α	F: 5′-AGCCCATGTTGTAGCAAACC-3′	134	94 °C for 2 min; 30 cycles of 94 °C 20s, 64 °C 2 min, 72 °C 30s: following by 72 °C for 7 min
	R: 5′-TGAGGTACAGGCCCTCTGAT-3′		

### Statistical Analysis

All experiments were performed in triplicate, and the results were reported as the mean ± SEM of triplicated experiments. Data were analyzed using the Student’s *t* test with two-tailed distribution and considered statistically significant at a *p* value ≤ 0.05.

## Results

### Survival of LAB in Simulated Gastrointestinal Solution

**Fig. 1:**
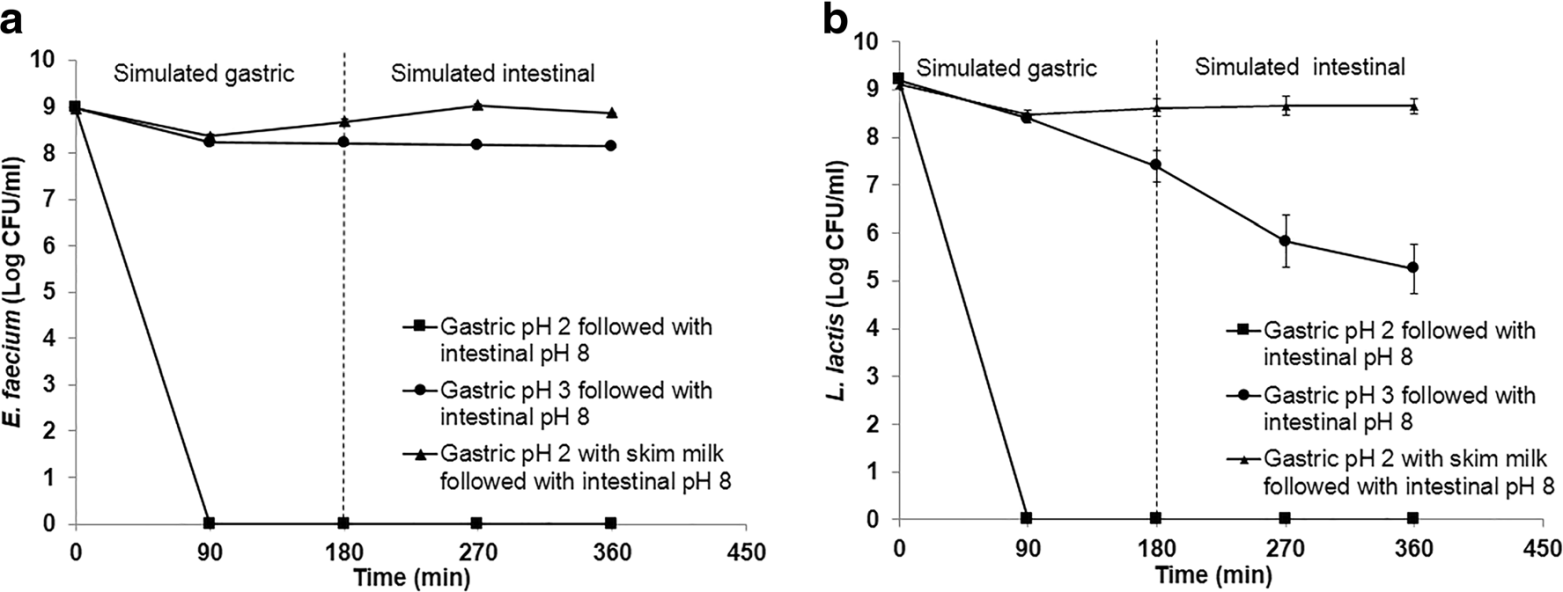
Cell viability (log CFU/ml) of *E. faecium* (**a**) and *L. lactis* (**b**) incubated in simulated gastric solution at pH 2 (square) and pH 3 (circle), followed by simulated intestinal solution at pH 8; and gastric solution at pH 2 mixed with skimmed milk (triangle), followed by intestinal solution at pH 8. Bars represent standard deviation values

Acid and bile salt tolerance are crucial properties for probiotic microorganisms which help them to survive through gastrointestinal tract. The survival of *E. faecium* and *L. lactis* in simulated gastrointestinal conditions was studied whether they can survive under acidic and alkaline condition. The result showed that the cell viability of *E. faecium* and *L. lactis* was drastically reduced during 90 min of incubation in simulated gastric solution at pH 2 (Fig. 1a, b). Both isolates survived in simulated gastric solution at pH 3 followed by intestinal solution at pH 8. However, *L. lactis* was more sensitive to these conditions, as its cell viability rapidly decreased compared to that of *E. faecium*. In addition, in the presence of skimmed milk added as a food matrix, both isolates subsequently demonstrated higher bacterial cell viability following incubation in simulated gastrointestinal solution.

### Adhesion Assay

**Fig. 2:**
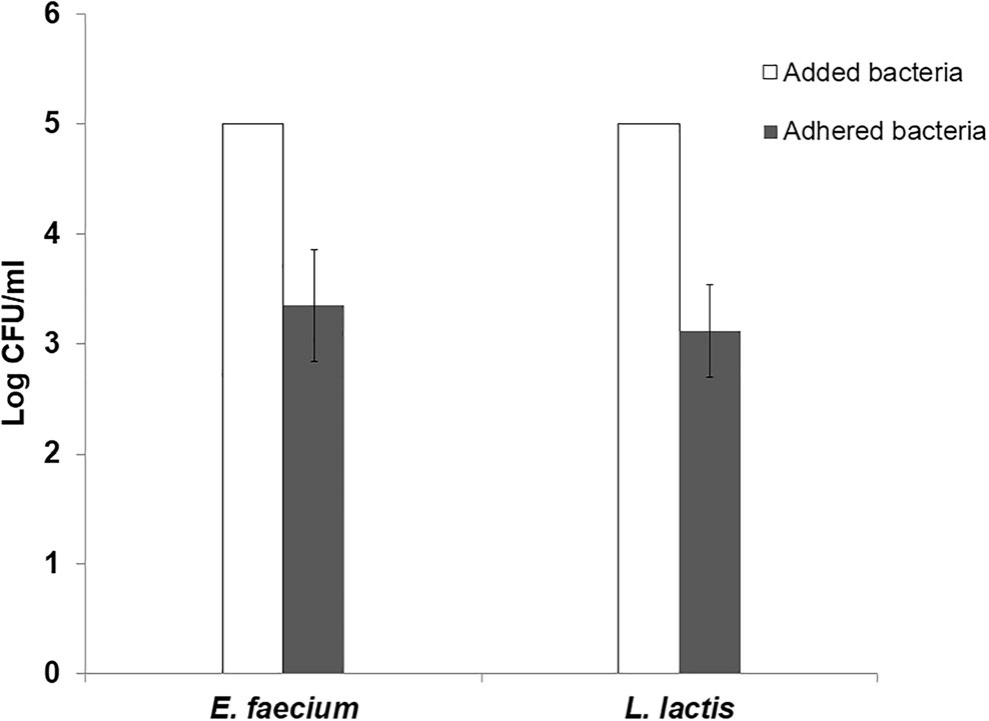
Adhesion ability of *E. faecium* and *L. lactis* to Caco-2 cells. The bacteria added to the Caco-2 cells vs. those which adhered are represented. Bars represent standard deviation values

The ability to adhere to intestinal epithelial cells is one of the most important properties for any probiotic bacteria in order to colonize in the gut mucosa. Caco-2 cells were used as a model of human colonic cells to study the adhesion properties of selected LAB. The result indicated that more than 60% of added *E. faecium* (61%) and *L. lactis* (67%) were able to adhere to Caco-2 cells (Fig. 2). There was no significant difference between the adhesive abilities of the isolates.

### Cytotoxicity Assay

**Fig. 3:**
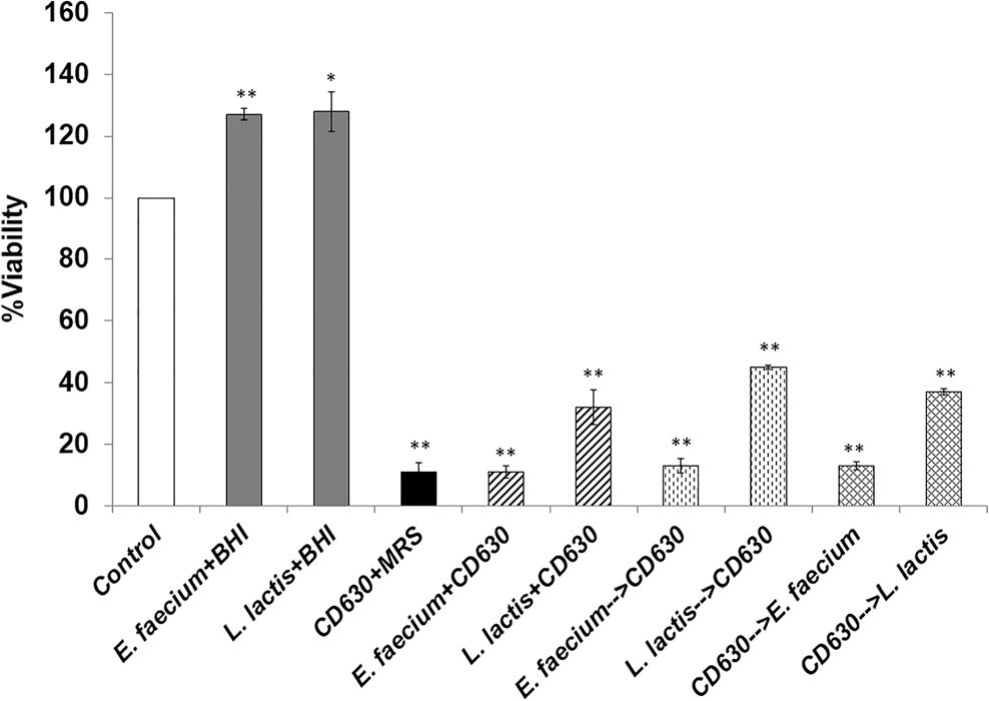
Cell viability of Caco-2 cells incubated with the NCFS of *E. faecium* or *L. lactis* combined with the CFS of CD630 for 24 h (co-incubation: *E. faecium*+CD630, *L. lactis*+CD630). The Caco-2 cells were pre-incubated with the NCFS of *E. faecium* or *L. lactis* for 2 h, followed by the addition of CFS of CD630 for 24 h (protection: *E. faecium* → CD630, *L. lactis* → CD630) and vice versa (prevention: CD630 → *E. faecium*, CD630 → *L. lactis*). Results are expressed as means ± SEM of triplicate experiments. The statistically significant difference from the control with the *p* value ≤ 0.05 and ≤ 0.01 was indicated by one asterisk (*) and double asterisks (**), respectively

Evaluation of bacterial toxicity is necessary when those bacterial strains are considered to be ingested as probiotics. Any probiotic strains should not be harmful to the cells. Therefore, the NCFS from *E. faecium* and *L. lactis* and the CFS of CD630 were tested for their toxicity toward Caco-2 cells using an MTT assay. The results demonstrated that the NCFS of *E. faecium* and *L. lactis* were not toxic to Caco-2 cells, while the CFS of CD630 exhibited significant toxicity at increasing total protein concentrations (data not shown). The cytotoxicity of the NCFS of each LAB coincubated with the CFS of CD630 in different incubation patterns was also studied. The results demonstrated that the NCFS of *L. lactis* significantly improved Caco-2 cell viability compared to that of the NCFS of *E. faecium* when incubated with the CFS of CD630 (Fig. 3). Changes in the incubation patterns (coincubation, protection, and prevention) did not significantly affect Caco-2 cytotoxicity when mixed with NCFS of LAB isolates with CFS of CD630.

### Immunofluorescent Imaging of Tight Junction in Caco-2 Cell

**Fig. 4:**
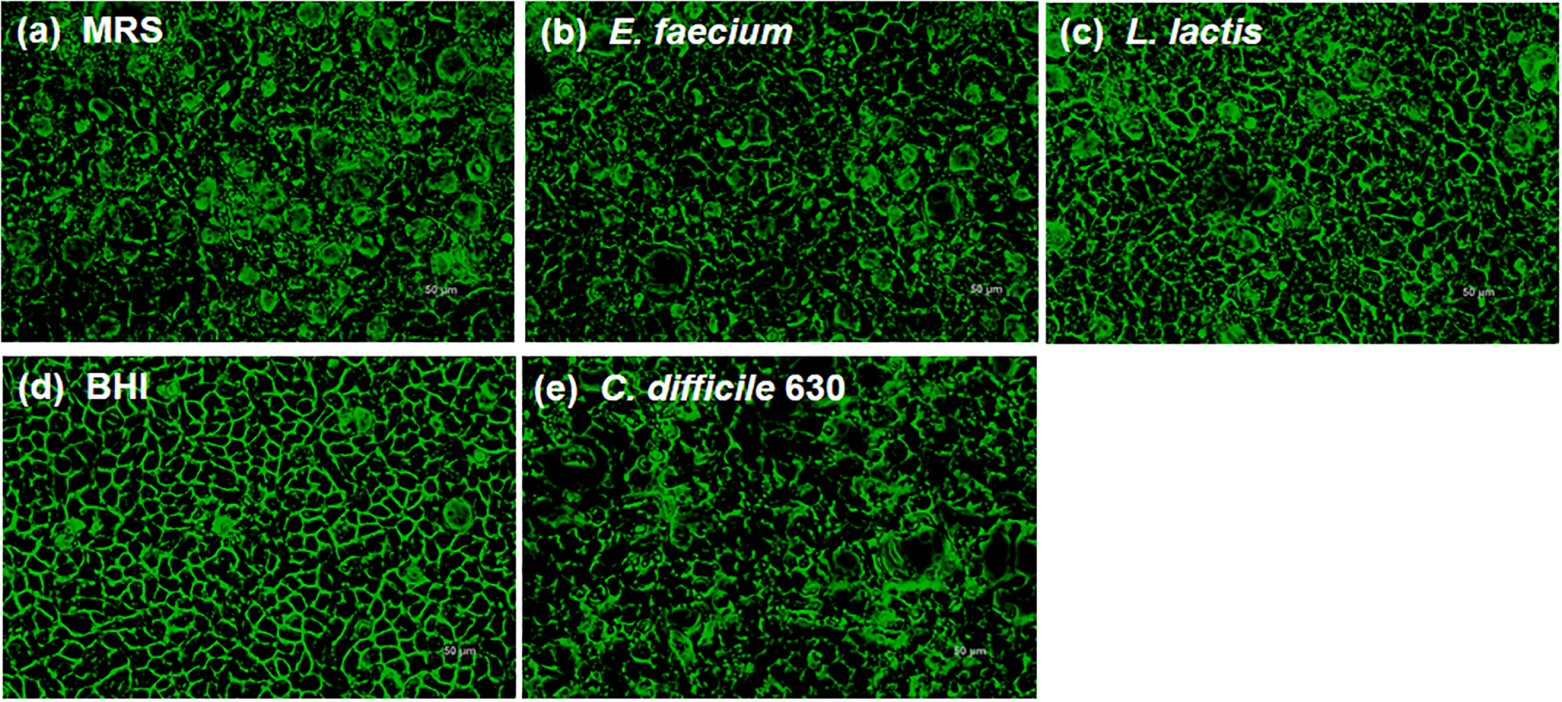
Immunofluorescence staining of occludin in Caco-2 cells incubated with MRS (**a**), the NCFS of *E. faecium* (**b**), the NCFS of *L. lactis* (**c**), BHI (**d**), and the CFS of CD630 (**e**)

Tight junction is a crucial component of an epithelial barrier. Microorganisms have to disrupt proteins in tight junction in order to penetrate the epithelial cells. Hence, the effects of the NCFS of *E. faecium* and *L. lactis* and the CFS of CD630 on tight junctions were observed by immunofluorescence staining of occludin, a transmembrane protein located at the tight junctions. The results demonstrated that the presence of MRS broth as a control treatment showed a morphological effect on Caco-2 cells (Fig. 4a). The NCFS of *E. faecium* and *L. lactis* showed minimal effects on tight junctions, as the structures of most cell membranes were still intact (Fig. 4b, c). Not surprisingly, compared to that of BHI broth as a control treatment (Fig. 4d), the CFS of CD630 affected the integrity of tight junctions by disrupting the structure of the cell membrane (Fig. 4e).

### Effect of LAB and CD630 on Immunomodulation

**Fig. 5:**
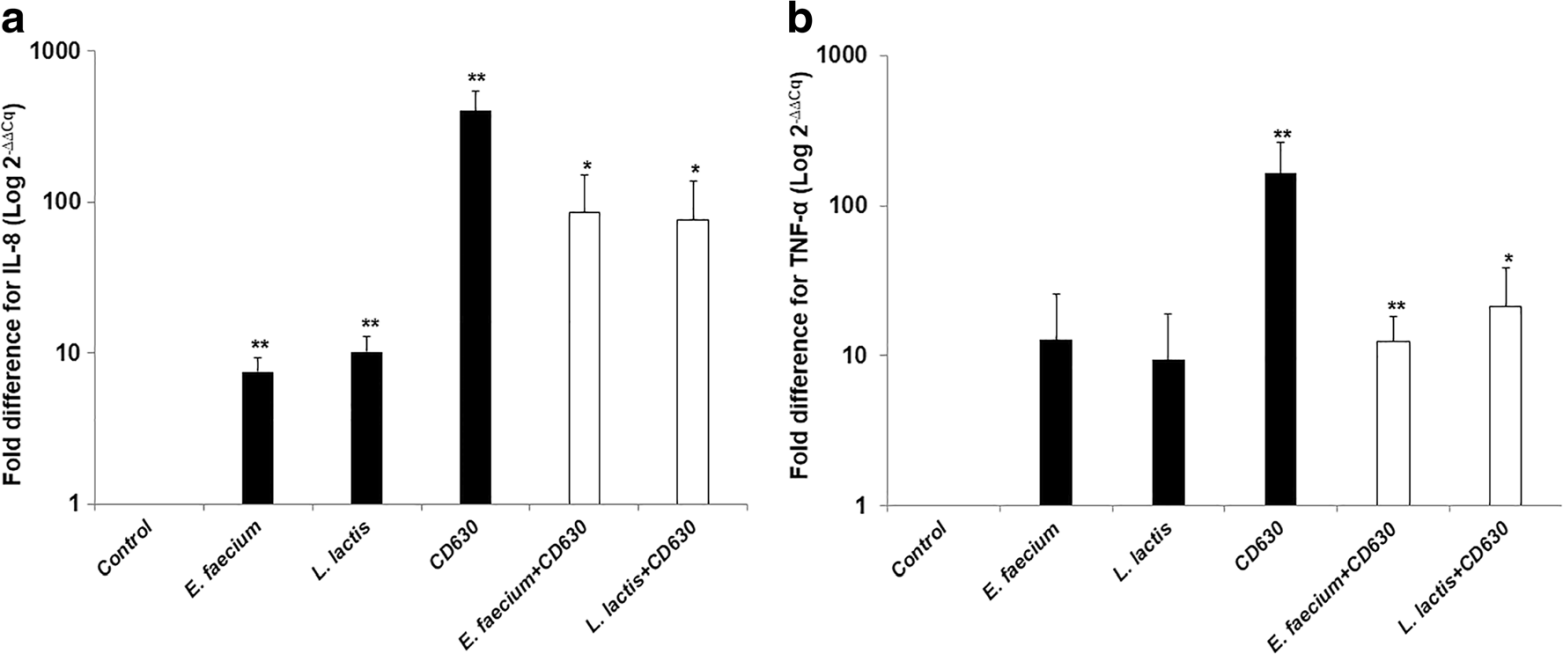
Fold differences in pro-inflammatory cytokine expression; IL-8 (**a**) and TNF-α (**b**) after incubation with the NCFS of *E. faecium*, *L. lactis*, and the CFS of CD630. Results are expressed as means ± standard deviation for duplicate experiments. The statistically significant difference from the control with the *p* value ≤ 0.05 and ≤ 0.01 was indicated by one asterisk (*) and double asterisks (**), respectively

The level of pro-inflammatory cytokine expression in the Caco-2 cells, such as IL-8 and TNF-α, was investigated using RT-qPCR. Optimum conditions for β-actin, IL-8, and TNF-α expressions were achieved via PCR (data not shown). The results indicated that IL-8 and TNF-α were expressed at a low level when Caco-2 cells were incubated with the NCFS of *E. faecium* and *L. lactis*, while the CFS of CD630 significantly increased the level of IL-8 and TNF-α expressions in Caco-2 cells (Fig. 5a, b). The effects of the NCFS of either *E. faecium* or *L. lactis* coincubated with the CFS of CD630 were also observed. The presence of the NCFS of *E. faecium* and *L. lactis* significantly reduced the expression levels of IL-8 and TNF-α caused by the CFS of CD630.

## Discussion

Our findings indicate that *E. faecium* and *L. lactis* isolated from Thai fermented sausages possess desirable probiotic properties. Both were resistant to simulated gastrointestinal solution, one of the most common probiotic properties. Our results are in agreement with those of previous studies, which reported that some LAB isolated from vaginal microflora, as well as *E. mundtii* 50H isolated from traditional dairy, were tolerant of low pH, bile salts, and simulated digestive conditions in vitro [15, 16]. *L. lactis* isolated from sea pineapple kimchi also showed high resistance to acidic and bile salt conditions [17].

Following LAB survival through the stomach and small intestine, those bacteria should be able to adhere to colon epithelial cells and initiate colonization. Most LAB have high adhesive abilities, and their adhesion to Caco-2 cells was recently reported [15–17]. In the present study, more than 60% of added *E. faecium* and *L. lactis* isolates were able to adhere to Caco-2 cells. Previous research suggests that various strains of *Lactobacillus* and *Bifidobacterium* possess high adhesive ability to human intestinal cell lines and that proteinaceous components in their spent culture broth also increase adhesion ability [18–20]. However, the adhesive property of LAB may vary from strain to strain, as each strain has different cell-surface molecules that play role in adhesion ability.

Any probiotic strains should be harmless to hosts. In the present study, we used the NCFS of LAB to study their cytotoxic effect on Caco-2 cells. We demonstrated that the NCFS of *E. faecium* and *L. lactis* isolates were not toxic to Caco-2 cells but rather increased Caco-2 cell viability. Cytotoxicity of the CFS of CD630 was evaluated as well, and the results demonstrated that the CFS of CD630 was highly toxic to Caco-2 cells. Furthermore, the NCFS of *E. faecium* and *L. lactis* facilitated cell viability and demonstrated cell protection abilities when coincubated with the CFS of CD630. Recent studies support our findings that LAB can help protect cells from clostridial toxins. In one such study by Eden et al., *L. rhamnosus* GG was cocultured with *C. difficile* at different concentrations, and the CFS of cocultured bacteria were incubated with Vero cells. They found that *L. rhamnosus* GG reduced the cytotoxic effects of clostridial toxins compared to pure *C. difficile* supernatant [7].

Most pathogens cause the disruption of epithelial barriers, including *C. difficile*. The toxin A (TcdA) influenced F-actin and enhanced tight junction permeability in the T84 cell line [21]. The T84 cell line was treated with *C. difficile* reference toxin A (TcdA-10463) and toxin B (TcdB-10463), and both toxins altered tight junction proteins, which subsequently increased paracellular permeability causing cell death [22]. There is considerable evidence that LAB enhance the integrity of epithelial cells and as such protect these cells from pathogenic bacterial invasion. A previous study by Anderson et al. demonstrated that 19 tight junction-related genes were altered in response to *L. plantarum* MB452, and the intensity of the immuno-stained ZO-1, ZO-2, and occludin appeared higher in Caco-2 cells treated with *L. plantarum* MB452 [23]. In another study, the probiotic mixture VSL#3 was used to treat a dextran sodium sulfate (DSS)-induced colitis mouse model. Results demonstrated that the VSL#3 protected the epithelial barrier by maintaining the tight junction protein expression in this murine model [24]. Using immunofluorescence staining, we achieved parallel results indicating that NCFS of *E. faecium* and *L. lactis* did not affect the integrity of tight junction proteins in Caco-2 cells. In contrast, the CFS of CD630 observably damaged tight junction proteins, as the disruption of cell membranes was observed under a fluorescence microscope.

Another critical property of probiotics is immunomodulation of the host cell. Various strains of LAB are capable of activating immune responses against pathogens. During infection, pro-inflammatory cytokines are released and subsequently trigger cell inflammation. Injection of TcdA and TcdB into human intestinal xenografts caused histopathological changes and acute mucosal inflammation, and both toxins elevated levels of IL-8 gene expression and protein production [25]. Recent findings have demonstrated that some LAB alleviate inflammation. In one study by Tian et al., *E. faecium* HDRsEf1 live cells and CFS were used to treat porcine epithelial cells from the jejunum, and their anti-inflammatory effects against *Escherichia coli* (ETEC) were observed. Both live cells and CFS decreased the level of IL-8 gene expression [26]. Luerce et al. demonstrated that the supernatant of *L. lactis* NCDO2118 reduced the production of IL-8 and TNF-α in Caco-2 cells. Administration of these bacteria also ameliorated the histological damage in the colon of a DSS-induced colitis mouse model [27]. We obtained similar data that the NCFS of *E. faecium* and *L. lactis* reduced the level of IL-8 and TNF-α when coincubated with the CFS of CD630 in Caco-2 cells. As a result, *E. faecium* and *L. lactis* isolates exhibited anti-inflammatory effects against CD630.

In conclusion, *E. faecium* and *L. lactis* isolated from Thai fermented sausages are suitable candidates for use as probiotics, exhibiting such desirable qualities as high adhesion to and low cytotoxicity toward Caco-2 cells from the human intestinal epithelium, as well as cytoprotective and anti-inflammatory properties. These bacteria could be used as an alternative treatment to prevent CDI. However, additional study is required to determine the effect of using live *E. faecium* and *L. lactis* isolates in vivo.
